# Pediatric Rhabdomyosarcomas: Three-Dimensional Radiological Assessments after Induction Chemotherapy Predict Survival Better than One-Dimensional and Two-Dimensional Measurements

**DOI:** 10.3390/cancers12123808

**Published:** 2020-12-17

**Authors:** Giovanna Orsatti, Carlo Morosi, Chiara Giraudo, Alessia Varotto, Filippo Crimì, Miriam Bonzini, Marta Minotti, Anna Chiara Frigo, Ilaria Zanetti, Stefano Chiaravalli, Michela Casanova, Andrea Ferrari, Gianni Bisogno, Roberto Stramare

**Affiliations:** 1 Radiology Institute, Department of Medicine, University of Padova, 35121 Padova, Italy; giovanna.orsatti@gmail.com (G.O.); chiara.giraudo@unipd.it (C.G.); ale.varotto86@gmail.com (A.V.); roberto.stamare@unipd.it (R.S.); 2 Department of Radiology, Fondazione Istituto di Ricovero e Cura a Carattere Scientifico (IRCCS) Istituto Nazionale dei Tumori, 20133 Milan, Italy; carlo.morosi@istitutotumori.mi.it (C.M.); miriam.bonzini@istitutotumori.mi.it (M.B.); marta.minotti@istitutotumori.mi.it (M.M.); 3 Unit of Biostatistics, Epidemiology and Public Health, Department of Cardiac, Thoracic, Vascular Sciences and Public Health, University of Padova, 35121 Padova, Italy; annachiara.frigo@unipd.it; 4 Haematology Oncology Division, Department of Women’s and Children’s Health, University of Padova, 35121 Padova, Italy; ilaria.zanetti@unipd.it (I.Z.); gianni.bisogno@unipd.it (G.B.); 5 Pediatric Oncology Unit, Fondazione Istituto di Ricovero e Cura a Carattere Scientifico (IRCCS) Istituto Nazionale dei Tumori, 20133 Milan, Italy; stefano.chiaravalli@istitutotumori.mi.it (S.C.); michela.casanova@istitutotumori.mi.it (M.C.); andrea.ferrari@istitutotumori.mi.it (A.F.)

**Keywords:** pediatric, imaging, rhabdomyosarcoma, response assessment

## Abstract

**Simple Summary:**

The prognostic significance of assessing radiological response after induction therapy for pediatric rhabdomyosarcoma (RMS) is still a matter of debate. In this retrospective study conducted at two centers on 66 non-metastatic RMS patients, we investigated the prognostic value of four radiological methods for measuring tumor response: 1D-RECIST (Response Evaluation Criteria in Solid Tumors), 2D-WHO (World Health Organization), 3D-EpSSG (European pediatric Soft tissue sarcoma Study Group) and 3D-Osirix. Patients were classified with each method as responders or non-responders based on the corresponding therapeutic cutoffs. Five-year event-free survival (5yr-EFS) was significantly longer for responders than for non-responders with all four methods, but the 3D assessments discriminated between the two groups better than 1D-RECIST or 2D-WHO. Inter-method agreement was excellent for 3D-EpSSG and 3D-Osirix, and moderate for the other comparisons. Inter-observer agreement was excellent with all methods except the 2D-WHO. Early tumor response was a significant prognostic factor in RMS, and the 3D-EpSSG and 3D-Osirix methods were better predictors of therapeutic response than the 1D-RECIST or 2D-WHO measurements.

**Abstract:**

Radiological response to neoadjuvant chemotherapy is currently used to assess the efficacy of treatment in pediatric patients with rhabdomyosarcoma (RMS), but the association between early tumor response on imaging and survival is still controversial. The aim of this study was to investigate the prognostic value of assessing radiological response after induction therapy in pediatric RMS, comparing four different methods. This retrospective, two-center study was conducted on 66 non-metastatic RMS patients. Two radiologists measured tumor size on pre- and post-treatment magnetic resonance (MR) or computed tomography (CT) images using four methods: considering maximal diameter with the 1D-RECIST (Response Evaluation Criteria in Solid Tumors); multiplying the two maximal diameters with the 2D-WHO (World Health Organization); multiplying the three maximal diameters with the 3D-EpSSG (European pediatric Soft tissue sarcoma Study Group); obtaining a software-assisted volume assessment with the 3D-Osirix. Each patient was classified as a responder or non-responder based on the proposed thresholds for each method. Tumor response was compared with survival using Kaplan–Meier plots, the log-rank test, and Cox’s regression. Agreement between methods and observers (weighted-κ) was also calculated. The 5-year event-free survival (5yr-EFS) calculated with the Kaplan–Meier plots was significantly longer for responders than for non-responders with all the methods, but the 3D assessments differentiated between the two groups better than the 1D-RECIST or 2D-WHO (*p*_1D-RECIST_ = 0.018, *p*_2D-WHO_ = 0.007, *p*_3D-EpSSG_ and *p*_3D-Osirix_ < 0.0001). Comparing the 5yr-EFS of responders and non-responders also produced adjusted hazard ratios of 3.57 (*p* = 0.0158) for the 1D-RECIST, 5.05 for the 2D-WHO (*p* = 0.0042), 14.40 for the 3D-EpSSG (*p* < 0.0001) and 11.60 for the 3D-Osirix (*p* < 0.0001), indicating that the volumetric measurements were significantly more strongly associated with EFS. Inter-method agreement was excellent between the 3D-EpSSG and the 3D-Osirix (κ = 0.98), and moderate for the other comparisons (0.5 < κ < 0.8). The 1D-RECIST and the 2D-WHO tended to underestimate response to treatment. Inter-observer agreement was excellent with all methods (κ > 0.8) except for the 2D-WHO (κ = 0.7). In conclusion, early tumor response was confirmed as a significant prognostic factor in RMS, and the 3D-EpSSG and 3D-Osirix methods predicted response to treatment better than the 1D-RECIST or 2D-WHO measurements.

## 1. Introduction

Soft tissue sarcomas account for approximately 7% of all childhood malignancies, and 1% of cancers in adults [1]. Rhabdomyosarcoma (RMS) is the most common soft tissue sarcoma in pediatric age, accounting for 50% of all cases in children, adolescents and young adults, with an annual incidence of 4.5 per million population under the age of 20 [1,2]. It is an aggressive tumor deriving from primitive mesenchymal cells committed to developing into striated muscles [1]. In two in every three cases it occurs in the head and neck area, but any body part may be affected [2]. In the past 30 years, the cure rate for RMS has improved dramatically, from 25% to around 70%, thanks to multidisciplinary and risk-adapted treatment approaches developed by international cooperative groups, which involve surgery, chemotherapy and radiotherapy [2]. Most patients are diagnosed with unresectable lesions, however, and initially receive neoadjuvant chemotherapy [3]. Early response to chemotherapy, assessed from changes in tumor size on computed tomography (CT) or magnetic resonance imaging (MRI), has recently emerged as a significant prognostic factor in many pediatric cancers, including Ewing sarcoma, osteosarcoma, neuroblastoma, and Hodgkin’s lymphoma [4,5,6,7,8]. Assuming that response to induction therapy is an outcome predictor in RMS as well, the European pediatric Soft-Tissue Sarcoma Study Group (EpSSG) currently tailors subsequent treatments based on early radiological response, including chemotherapy-induced changes in patients identified as poor responders [2]. The prognostic role of initial tumor response and the most appropriate way to measure tumor dimensions are still debated in the literature, however [9,10,11,12,13]. Regarding the latter aspect, single-axis measurements (Response Evaluation Criteria in Solid Tumors (RECIST)) or 2D (World Health Organization (WHO)) guidelines are usually applied in adults [14,15,16]. These methods may be representative of tumor size in the case of spherical masses, but most RMS grow and shrink asymmetrically [12,17,18]. The EpSSG and the North American Children’s Oncology Group (COG) have proposed a volumetric method based on measuring the three maximal diameters of a tumor [2,19]. A few studies have compared the EpSSG and RECIST criteria applied to cases of RMS, and neither method has so far shown a better performance in terms of reliability and/or prognostic value [12,20], whereas 3D measurements have proved better predictors of outcome in pediatric Ewing sarcoma [19]. Several authors have also investigated the reproducibility of a software-assisted volumetric method for measuring tumor extent in various adult and pediatric cancers, but their findings have been controversial [21,22,23,24,25,26]. 

To the best of our knowledge, no previous studies compared different methods for assessing the prognostic value of initial tumor response in RMS. The purpose of our study was therefore to compare four radiological methods (1D, 2D, 3D EpSSG, and software-assisted 3D) for assessing early response in localized RMS, and to identify the method that best predicts clinical outcome.

## 2. Results

### 2.1. Patient Characteristics

A total of 49 of the 115 patients with non-metastatic RMS identified at our centers from January 2005 to December 2016 did not meet our inclusion criteria, so imaging findings were examined for 66 patients. This sample had a mean age ± standard deviation of 7.8 years ± 6.1, and a median age of 6.1 years (range 0.01–22.5). A total of 56 cases were analyzed on MR images and 10 on CT scans. The median time elapsing from diagnosis to early tumor response assessment was 2.4 months (range: 1.8–6.0).

The main characteristics of the patients and their disease are summarized in Table 1.

In terms of risk stratification, 13 of the 66 patients were assigned to a standard risk group, 49 to a high-risk group, and 4 to a very high-risk group. As induction chemotherapy, 45 patients received three cycles of IVA (ifosfamide, vincristine, actinomycin), and 21 patients received three cycles of IVADo (ifosfamide, vincristine, actinomycin and doxorubicin). Table 1 shows details of the chemotherapy regimens by risk group. A total of 29 patients had secondary surgery, while 37 did not, either because the tumor site made it unfeasible or because they had achieved a complete response. Sixty-one patients had radiotherapy as part of their treatment.

The median follow-up was 63.9 months (range 19.7–149.1). Overall, 20 patients experienced an event affecting survival, which involved: disease progression in six cases; both local and metastatic relapses in two; metastatic relapses alone in five; second malignancies in three (osteosarcoma, acute myeloid leukemia and inflammatory myofibroblastic tumor). A total of 15 patients died, 14 of a RMS relapse and one of secondary osteosarcoma.

### 2.2. Agreement between Observers and Methods for Tumor Response Assessment

Inter-observer agreement regarding response was excellent for the 1D-RECIST, 3D-EpSSG and 3D-Osirix methods (κ = 0.83, 0.86 and 0.95, respectively), and substantial for the 2D-WHO method (κ = 0.70).

**Figure 1:**
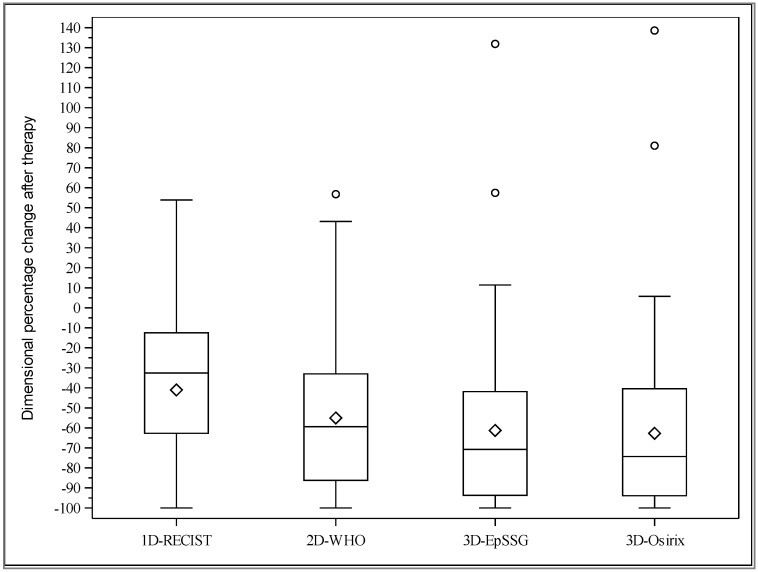
Box plots of percentage changes in tumor size across patients for the four methods. The box plot fences represent the first and third quartiles. Whiskers cover the extent of the data on 1.5 x the interquartile range. The line bisecting the box represents the median. The diamond inside the box represents the mean. The circles represent the outlier values.

The distribution of the percentage reduction in tumor size across patients was highly skewed with all methods, and the skewness increased with larger dimensional measurements. The percentage reduction was greater with the volumetric methods: the median percentage decrease in tumor size after induction therapy was 32.5% with the 1D-RECIST, 59.4% with the 2D-WHO, 70.7% with the 3D-EpSSG, and 74.3% with the 3D-Osirix (Figure 1).

**Figure 2:**
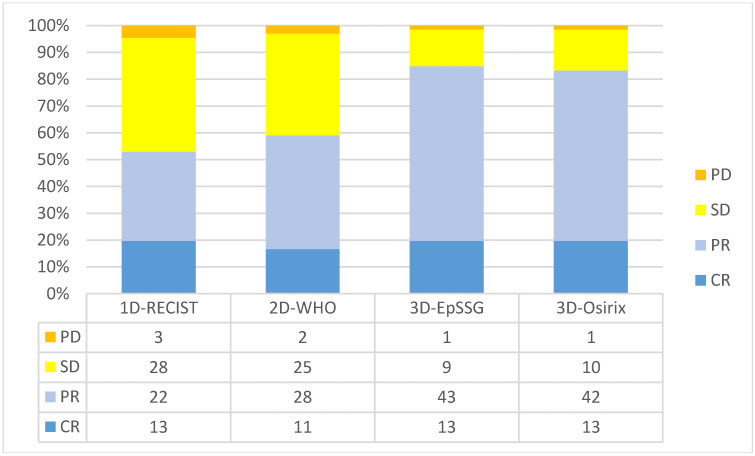
Bar plot showing the four categories of early response assessed with the four methods. CR = complete response; PR = partial response, SD = stable disease, PD = progressive disease.

A total of 35 patients (53%) were classified as responders with the 1D-RECIST, 39 (59%) with the 2D-WHO, 56 (85%) with the 3D-EpSSG, and 55 (83%) with the 3D-Osirix (Figure 2).

Inter-method agreement was excellent for the two volumetric methods, with a κ value of 0.97, and substantial for the 1D-RECIST and the 2D-WHO, for the 2D-WHO and the 3D-EpSSG, and for the 2D-WHO and the 3D-Osirix, with κ values of 0.60, 0.60 and 0.62, respectively. Agreement was moderate for the other comparisons, with κ values <0.6 but >0.5 (Table 2).

The disagreement between the 1D-RECIST and the 3D-EpSSG, between the 1D-RECIST and the 3D-Osirix, between the 2D-WHO and the 3D-EpssG, or between the 2D-WHO and the 3D-Osirix would have led to a different therapeutic approach for 22, 21, 18, and 17 patients, respectively. In all cases, this would have been due to the 3D methods shifting patients from the non-responder to the responder group.

### 2.3. Comparison between Radiological Response and Survival

**Figure 3:**
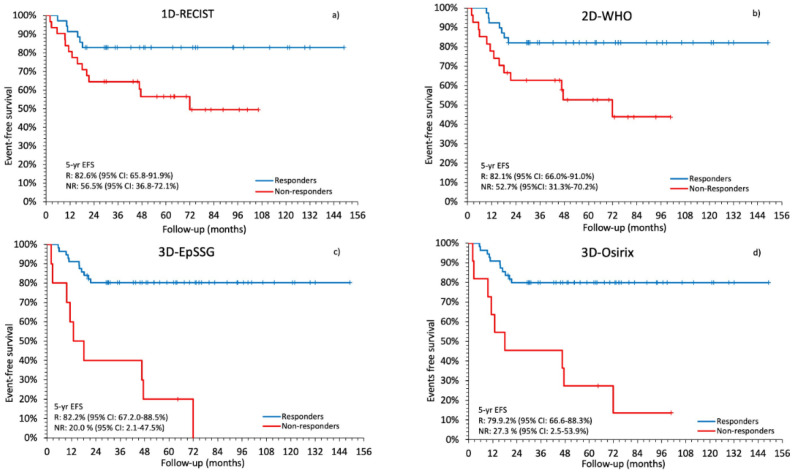
Kaplan–Meier plots comparing the event-free survival (EFS) predicted with each method for responders and non-responders. The log-rank test showed the following *p* values: 0.0181 with the 1D-RECIST (**a**); 0.0073 with the 2D-WHO (**b**); <0.0001 with the 3D-EpSSG (**c**) and the 3D-Osirix (**d**).

In our analysis, image-based tumor response was a significant predictor of event-free survival (EFS) with all four types of measurement. Five-year EFS was always significantly longer in responders than in non-responders: 82.6% vs. 56.5% (*p* = 0.0181) with the 1D-RECIST; 82.1% vs. 52.7% (*p* = 0.0073) with the 2D-WHO; 82.2% vs. 20.0% (*p* < 0.0001) with the 3D-EpSSG; 79.9% vs. 27.3% (*p* < 0.0001) with the 3D-Osirix. In the non-responder group, the 3D-EpSSG and the 3D-Osirix predicted a much shorter 5-yr EFS than the 1D-RECIST and the 2D-WHO, with no noteworthy overlap in the corresponding 95% confidence intervals (CIs) (Figure 3a–d).

**Figure 4:**
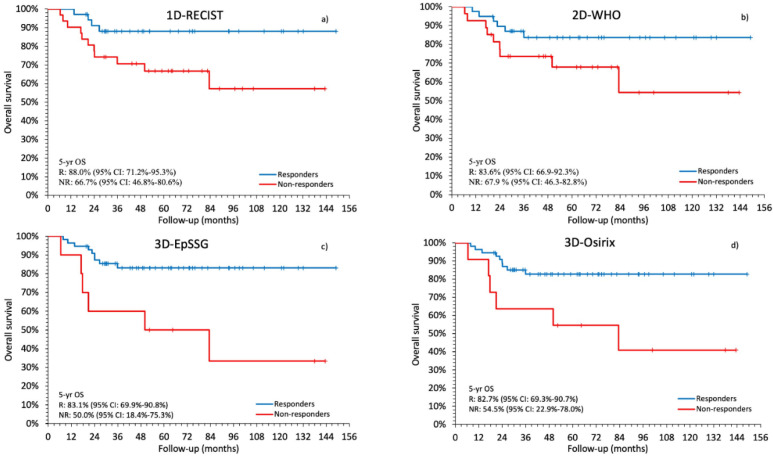
Kaplan-Meier plots comparing overall survival (OS) predicted with each method for responders and non-responders. The log-rank test showed the following *p* values: 0.0304 with the 1D-RECIST (**a**); 0.0794 with the 2D-WHO (**b**); 0.0025 with the 3D-EpSSG (**c**); 0.0087 with the 3D-Osirix (**d**).

Radiological tumor response also predicted overall survival (OS) (Figure 4a–d) using three of the four methods (*p*_1D-RECIST_ = 0.0304, *p*_2D-WHO_ = 0.0794, *p*_3D-EpSSG_ = 0.002 and *p*_3D-Osirix_ = 0.0087).

Univariate Cox’s proportional hazards regression analysis demonstrated that early response, expressed as a categorical variable, was a significant prognostic factor for EFS, with no clear differences between the four methods: there was a considerable overlap in the hazard ratios (HRs) and 95% CIs, and the corresponding Uno’s values were comparable (Table 3). Assessed categorically, tumor response was also a significant predictor of OS with all methods except for the 2D-WHO.

Early radiological response, expressed as a percentage reduction in tumor size, was a significant factor in predicting EFS, regardless of the method used, but never a significant predictor of OS (Table 4). 

In the multivariate analysis (Table 5), the adjusted HRs were higher overall than the unadjusted values for all methods. For EFS, the adjusted HRs were considerably higher with the 3D-EpSSG and the 3D-Osirix than with the 1D-RECIST or the 2D-WHO, with no remarkable overlap in the corresponding 95% CIs. For OS, the adjusted HRs obtained with the 2D-WHO was not significant (p = 0.0849). There were significant associations between tumor size and site (*p* < 0.0001), between histology and age at diagnosis (*p* = 0.0038), and between histology and tumor response assessed with the 2D-WHO (*p* = 0.043). No significant associations emerged between the other prognostic factors.

## 3. Discussion

The main finding emerging from this study is that image-based early tumor response was significantly associated with EFS, whatever the radiological method used to assess tumor size. That said, the 3D-EpSSG and the 3D-Osirix were better predictors of 5-year EFS than the 1D-RECIST or the 2D-WHO, when tumor response was assessed categorically. 

Our results are consistent with two previous reports of early response, proving an important factor for predicting survival in patients with localized RMS. In a study by Dantonello et al. [13], the prognosis was significantly better for responders than for non-responders when response was assessed in terms of tumor volume reduction. Ferrari et al. [12] likewise found that a poor response to initial chemotherapy correlated with a poor prognosis, regardless of whether the 1D-RECIST or the 3D-EpSSG were used to measure response. These findings contrast with a recent analysis conducted by the International Society of Paediatric Oncology’s Malignant Mesenchymal Tumor Group and the COG, which concluded that early radiological response measured with the 2D-WHO was of no prognostic value [9,10,11]. Among the likely reasons for this diversity in the published findings, one concerns the fact that, in all previous studies, radiological response was measured by radiologists at each local institution without any central review, and this could have affected the results. Second, some authors excluded patients with progressive disease from their analysis, and this might explain a weaker association with clinical outcome [9,10,11].

Our data are also consistent with several studies conducted on other pediatric malignancies, in which initial response to chemotherapy was found to predict outcome in Ewing sarcoma [5], osteosarcoma [6], Hodgkin’s lymphoma [7], and neuroblastoma [8]. 3D measurements have proved more sensitive than the 1D-RECIST or the 2D-WHO in predicting clinical outcome in adult gastrointestinal stromal tumors [27], lung cancer [28] and pediatric Ewing sarcoma [19].

In our analysis, the 3D-EpSSG and the 3D-Osirix indicated a considerably shorter 5-year EFS for non-responders than the 1D-RECIST or the 2D-WHO, with higher EFS-related adjusted HRs (Figure 3 and Table 5). These results suggest that volumetric measurements better discriminate between the two response categories, achieving a stronger association with clinical outcome. That said, like Ferrari et al. [12], we found no significant differences between the four different methods’ prognostic value when early response was assessed in terms of percentage changes in tumor size. Although measuring tumor response as a continuous variable might increase the statistical power, it can prove difficult to adopt continuous measurements instead of cut-offs in clinical practice [9].

The second main finding of this study is that measuring more dimensions when assessing response on imaging findings enables a better classification of response. Volumetric methods classified more patients as responders than the 1D-RECIST or the 2D-WHO because tumor shrinkage tended to be quantified as greater when assessed in terms of volume than when assessed as a diameter or area. Assuming that tumors are generally considered as being more or less spherical in shape, this finding may be geometrically justified by the fact that changes in diameter are smaller than corresponding changes in cross-sectional area, which are in turn smaller than corresponding changes in volume. In short, a 30% decrease in diameter coincides with a 50% decrease in cross-sectional area, and a 65% reduction in volume [12,20]. In clinical practice, RMS are usually very irregular in shape, and far from spherical.

Judging from our data, the 1D-RECIST and the 2D-WHO tended to underestimate response to treatment, and this could have a strong impact on a patient’s therapeutic management. The RMS 2005 EpSSG protocol [2] requires at least a partial response after three cycles for first-line chemotherapy to continue, while patients responding less well are switched to a different second-line regimen. That is why assessments of radiological response must be highly sensitive in the early identification of non-responders, to avoid continuing ineffective therapies and support the adoption of more aggressive treatments in an effort to improve the prognosis. For our study sample, the chemotherapy regimens did not change substantially over the years, and any differences in patient treatment strategies were due largely to the patients’ classification by risk group at diagnosis, or by response after induction therapy. Nonetheless, we found that inter-method variability would have a significant impact on treatment decisions as a substantial number of patients classified as non-responders using the 1D-RECIST and the 2D-WHO would still benefit from first-line chemotherapy.

The computerized measurement of tumor volumes has the potential to provide the most accurate assessment of tumor burden, and has been considered as the reference standard for assessing response in Ewing sarcoma [19]. Software-assisted volume calculation has proved more accurate than the 1D-RECIST or the 2D-WHO in adult glioma [21,22,23,24], and been associated with less inter-observer variability than the 3D-EpSSG in assessing RMS [26].

In this study, no significant differences emerged between the 3D-EpSSG and the 3D-Osirix in terms of reliability and association with survival. Therefore, would not recommend adopting software-assisted volumetric assessments in clinical practice, given that they are more time-consuming (taking 5 to 10 min to delineate each volume of interest).

We need to acknowledge a few limitations of our study. First, there is the small size of the sample, which is due to the rarity of pediatric RMS. Second, there is the study’s retrospective nature: our analysis was conducted on images acquired over a 12-year period and, though all patients were included in the same consecutive international prospective clinical trials, there was no imaging protocol specifically for this study. Because of this lack of standardization, we were unable to analyze other radiological parameters, such as tumor necrosis, vascularity or cellularity on diffusion-weighted images (DWI), which are well-known prognostic indicators in various cancers, including sarcomas [29,30,31]. Another potential limitation of this study lies in that 10 patients were investigated with CT, which has a lower contrast resolution for assessing soft tissues than MRI, and this might have affected the accuracy of the tumor size assessments on CT scans.

Future prospective studies based on standardized MRI protocols could examine whether quantifying tumor necrosis, vascularization and restricted diffusion on DWI lends additional prognostic value to assessments of response to treatment based on 3D tumor measurements. 

## 4. Materials and Methods

### 4.1. Study Design

This retrospective study was performed at two tertiary pediatric oncology referral centers. We considered clinical data from the medical records of all patients (aged 0–21 years) diagnosed between January 2005 and December 2016, in accordance with the following inclusion criteria: a histologically confirmed diagnosis of RMS; no distant metastases; no previous surgery other than diagnostic incisional biopsy (Intergroup Rhabdomyosarcoma Study (IRS) post-surgical system group III: biopsy with gross residual disease); availability of radiological images (CT or MRI) of the primary tumor at diagnosis and at week 9, after completing three cycles of neoadjuvant chemotherapy). The exclusion criteria were as follows: (a) no measurable pre-treatment primary tumor on MR or CT images due to extensive diagnostic surgery or image degradation by artifacts; (b) post-treatment assessment before completing three cycles or after more than three cycles of chemotherapy; (c) extensive surgical resection or radiation therapy before completion of the neo-adjuvant chemotherapy.

The study was conducted in accordance with the Declaration of Helsinki and all patients or their parents/guardians gave their written informed consent to their data being used for research as part of their enrollment in the RMS 2005 protocol [2].

After the diagnostic work-up (which included CT and/or MRI of the primary tumor, chest CT scan, radionuclide bone scan or FDG-PET, bone marrow aspirates and biopsy), patients were assigned to a risk group according to the EpSSG classification. This classification considers six prognostic factors: histological subtype (unfavorable if alveolar); type of tumor resection at diagnosis (as defined by the IRS grouping system); primary tumor site (favorable = orbit, genitourinary (but not bladder or prostate), and head and neck (but not parameningeal)); nodal involvement; tumor size (unfavorable if >5 cm); patient’s age (unfavorable if ≥10 years) [32]. Based on the EpSSG RMS 2005 protocol, patients with macroscopic disease after initial surgery were treated with three cycles of chemotherapy followed by an assessment of their radiological response (at week 9), which oriented decisions regarding local therapy (surgery and/or radiotherapy). A total of six more cycles of chemotherapy, with or without six months of maintenance chemotherapy, were then administered [2]. 

### 4.2. Tumor Size Measurements

For the purposes of this study, all images were centrally and independently reviewed by two observers, one with 7 and the other with 10 years of experience in pediatric radiology. Tumor size was measured on CT or MR images at diagnosis and after three cycles of induction chemotherapy to assess tumor response.

CT scans were performed with either a Somatom Sensation 64 or a Somatom Definition Flash 128 (Siemens Healthcare, Erlangen, Germany). MR scans were performed with a 1.5 Tesla Achieva (Philips Healthcare, Best, The Netherlands) or a 1.5 Tesla Avanto (Siemens Healthcare, Erlangen, Germany), using phased array body coils, with patients in the supine position. For the 1.5 Tesla Achieva MR sequences, each series of images included: (1) axial T2-weighted turbo spin echo (echo time 80 ms; repetition time 4540 ms; slice thickness 5 mm); (2) T2-weighted spectral attenuated inversion recovery turbo spin echo (echo time 80 ms; repetition time 4870 ms; slice thickness 5 mm); (3) axial T1 turbo field echo (echo time 4.6 ms; repetition time 10 ms; flip angle 15°; slice thickness 5 mm); (4) diffusion-weighted images (echo time ms; repetition time ms; b value 50/400/800 s/mm^2^; slice thickness 5 mm); (5) pre-contrast 3D T1-weighted high-resolution isotropic volume excitation with axial, sagittal, and coronal reconstructions (echo time 2.4 ms; repetition time; 6.5 ms; FA 10°; slice thickness 3 mm); (6) contrast-enhanced 3D T1-weighted high-resolution isotropic volume excitation obtained after 60 s of intravenous administration of 0.2 mL/kg gadoteric acid (Dotarem, Guerbet, Roissy CdG Cedex, France), followed by a 20-mL saline flush, with axial, sagittal, and coronal reconstructions. The 1.5 Tesla Avanto MR protocol included: (1) axial T2-weighted turbo spin echo (echo time 105 ms; repetition time 1100 ms; slice thickness 5 mm); (2) axial fat-suppressed T2-weighted turbo spin echo (echo time 89 ms; repetition time 2540 ms; slice thickness 5 mm); (3) axial T1-weighted turbo spin echo (echo time 4.87 ms; repetition time 170 ms; slice thickness 5 mm) (4) diffusion-weighted images (echo time 72 ms; repetition time 5100 ms; b value 50/400/800/1000 s/mm^2^; slice thickness 5 mm); (5) pre-contrast fat-suppressed T1-weighted Dixon volumetric interpolated breath-hold examination with axial, sagittal, and coronal reconstructions (echo time 2.0 ms; repetition time; 6.0 ms; flip angle 10°; slice thickness 3 mm); (6) contrast-enhanced isotropic fat-suppressed T1-weighted volumetric interpolated breath-hold examination after 60 s of intravenous administration of 0.1 mL/kg gadobutrol (Gadovist, Bayer Pharma AG, Berlin, Germany), followed by a 20-mL saline flush, with axial, sagittal, and coronal reconstructions.

Additional sequences were acquired on the sagittal or coronal planes as necessary, on a case-by-case basis, depending on a tumor’s site and characteristics. In the case of very young patients or small-sized tumors, the slice thickness was reduced to 3 mm for all sequences.

The measurements were obtained on contrast-enhanced T1-weighted MR images or T2-weighted MR images (in this order of preference, and using the same sequence before and after chemotherapy), or on contrast-enhanced CT images. CT was considered only if MR images at diagnosis and/or after three cycles were unavailable. 

**Figure 5:**
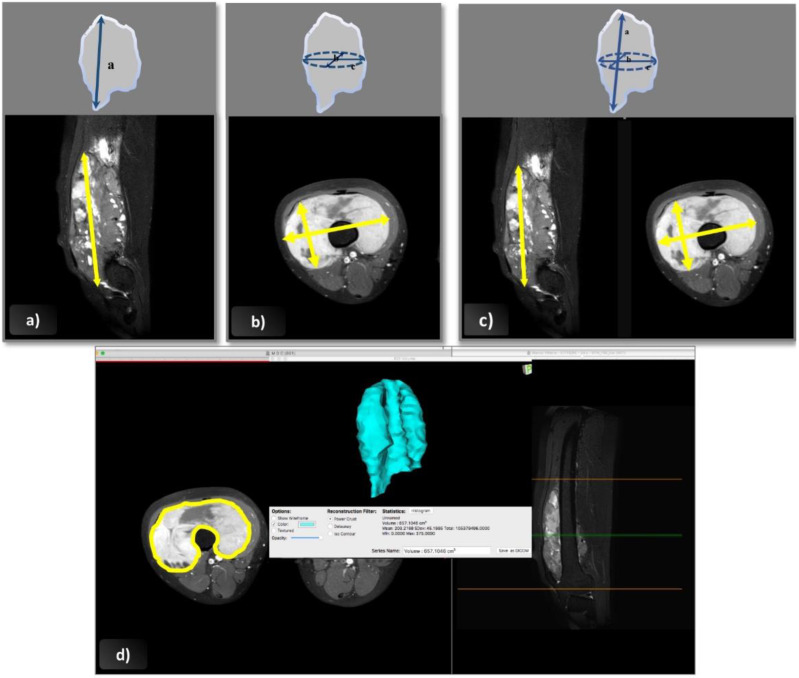
Tumor size measured as shown, with diagrams and axial and sagittal post-gadolinium fat-saturated T1w-MR images, according to the four methods: (**a**) 1D-RECIST (**b**) 2D-WHO, (**c**) 3D-EpSSG and (**d**) 3D-Osirix.

Tumor size was assessed using four methods: (1) the 1D-RECIST; (2) the 2D-WHO; (3) the 3D-EpSSG; (4) the 3D-Osirix (Figure 5).

1. Following the RECIST 1.1 guidelines, the longest tumor diameter was measured in any plane (axial, coronal, or sagittal) [25]. In the post-treatment assessment, the maximal diameter was measured in the same plane as at diagnosis, but not necessarily on the same slice level or in the same direction.
2. Cross-sectional area according to the WHO criteria was obtained from the product of the longest overall tumor diameter in the axial plane and the longest perpendicular diameter [14].
3. For the 3D-EpSSG assessment, a tumor’s three maximal diameters were assessed according to the guidelines in the EpSSG RMS 2005 protocol. The two maximal perpendicular diameters (*a* and *b*) were assessed in the axial plane on the section with the largest tumor surface area; the cranio-caudal dimension (*c*) was measured on sagittal or coronal images. Tumor volume was obtained with the following formula: *a* × *b* × *c* × π/6, which approximates the geometry of the tumor to an ellipsoid [2].
4. Software-assisted volume assessment was based on 2D cross-sectional measurements followed by volume rendering using Osirix software, version 5.6, 64 bit (Geneva, Switzerland). This software can calculate the volume of a solid lesion starting from a series of 2D regions of interest drawn by the operator. The tumor’s boundaries were outlined manually with the tool’s pencil, selecting one region of interest for each slice containing visible tumor on the axial plane, and avoiding the inclusion of any perilesional edema with the help of coronal and sagittal images. Then, the 3D tumor volume was reconstructed using the “multiplanar reformation compute volume” application, and the corresponding volume in cubic centimeters was recorded.

### 4.3. Tumor Response Assessment and Classification

Tumor response after induction chemotherapy was calculated as the percentage increase or decrease in diameter (1D-RECIST), surface area (2D-WHO) or volume (3D-EpSSG and 3D-Osirix) vis-à-vis tumor size at diagnosis. The percentage change in tumor size after treatment was ascertained according to the following formula:

((post-treatment value − pre-treatment value)/pre-treatment value) × 100

Tumor response was assessed on completion of the induction therapy (i.e., at week 9), and classified on the basis of the therapeutic response thresholds for the 1D-RECIST, 2D-WHO and 3D-EpSSG [19,20] (Table 6). The 3D-EpSSG classification of response was also applied to the 3D-Osirix volumetric measurements. To facilitate the comparison between the methods, the minor partial response (mPR) and very good partial response (VGPR) categories (exclusive to the EpSSG classification) were considered as subgroups of the partial response (PR) category.

Patients with a complete response (CR) or PR (including VGPR and mPR, where applicable) were classified as responders, and patients with stable disease (SD) or progressive disease (PD) as non-responders.

### 4.4. Statistical Analyses

Counts and percentages are reported for categorical variables, and means, standard deviations, medians and interquartile ranges for quantitative variables. Inter-observer and inter-method agreement for the classification of response were tested with the weighted Cohen’s κ coefficient and the 95% confidence interval (CI).

For inter-method agreement, cases of disagreement were solved by consensus.

Progressive disease, local and nodal relapse, metastases and the occurrence of a second malignancy were considered for EFS. For both EFS and OS, the follow-up time was calculated as the time elapsing from diagnosis to an event or death due to any cause, or latest follow-up. Patients with no event or death were censored at the time of their last reported contact. EFS and OS were estimated with the Kaplan–Meier method, and 5-year survival is reported with the 95% CI. The EFS and OS were compared between responders and non-responders using the log-rank test. The value of early radiological response in predicting EFS and OS was further assessed with a univariate Cox’s proportional hazards regression, in terms of both percentage changes in tumor size and classification of response (responders vs. non-responders). 

A multivariate Cox’s regression analysis was also applied to investigate whether the predictive value of the radiological response changed after adding the following prognostic factors identified in the literature as potential confounders [2,32,33]: histology, age, tumor size at diagnosis, tumor site, and nodal status. Fisher’s test was also used to examine the association between these variables, and between each variable and tumor response. 

The results of the Cox’s regression are expressed as *p*-values and HRs with 95% CIs. The accuracy of the radiological response in predicting survival, in terms of percentage change in tumor size and response category, was tested with the c-statistic expressed by Uno’s concordance probability (Uno) and its standard error. A *p*-value < 0.05 was considered indicative of statistical significance. All statistical analyses were performed with SAS 9.4 (SAS Institute Inc., Cary, NC, USA).

## 5. Conclusions

In conclusion, early tumor response proved to be a significant prognostic factor for patients with RMS using all four radiological methods considered here, but the volumetric assessments showed the strongest association with EFS. The 3D-EpSSG and the 3D-Osirix were equivalent, so we recommend using the less time-consuming 3D-EpSSG criteria in clinical practice. Their application could drive changes in the choice of treatment for patients identified as non-responders.

## Figures and Tables

**Table 1:** Patients’ clinical features.

Characteristic	N	%
*Sex*		
Male	34	51.5
Female	32	48.5
*Age (y)*		
Median (range)	6.1 (0.01–21)	
Mean ± sd	7.8 ± 6.1	
≤10	47	71.2
>10	19	28.8
*Tumor Site*		
Orbit	6	9.1
Head and neck parameningeal	28	42.4
Head and neck non parameningeal	3	4.6
Genitourinary, bladder or prostate	8	12.1
Genitourinary, non-bladder or prostate	1	1.5
Extremities	7	10.6
Other	13	19.7
*Histology*		
Embryonal	49	74.2
Alveolar	16	24.3
Not otherwise specified	1	1.5
*Tumor Size at Diagnosis*		
Diameter ≤ 5 cm	42	
Diameter >5 cm	24	
Median maximal diameter (iq range), cm	5.85 (1.58–20.1)	
Median cross sectional area (iq range), cm^2^	17.01 (1.24–126)	
Median 3d-epssg volume (iq range), cm^3^	41.09 (2.58–950.1)	63.6
Median 3d-osirix volume (iq range), cm^3^	39.42 (2.41–927.0)	36.4
*Tumor Size after Treatment*		
Median maximal diameter (iq range), cm	3.17 (0–22.0)	
Median cross sectional area (iq range), cm^2^	5.03(0–128.3)	
Median 3d-epssg volume (iq range), cm^3^	7.18 (0–1441.5)	
Median 3d-osirix volume (iq range), cm^3^	7.09 (0–1500.4)	
*T Status*		
T1 (without local invasion)	19	28.8
T2 (with local invasion)	47	71.2
*N Status*		
N0 (without regional nodal metastases)	53	80.3
N1 (regional nodal metastases)	12	18.2
Unknown	1	1.5
*Risk Group*		
Standard	13	19.7
High	49	74.2
Very High	4	6.1
*Chemotherapy Regimen*		
9 iva	25	37.9
9 iva + *6 vnl/cpm*	11	16.7
4 ivado + 5 iva	8	12.1
4 ivado + 5 iva + 6 vnl/cpm	13	19.7
4 iva + 5 topo/carbo	7	10.6
4 iva *	2	3
*Delayed Surgery*		
Yes	29	43.9
No	37	56.1
*Radiotherapy*		
Yes	61	92.4
No	5	7.6

IVA = ifosfamide, vincristine, actinomycin; VNL = vinorelbine; IVADO = ifosfamide, vincristine, actinomycin, doxorubicin; CPM = cyclophosphamide; TOPO = topotecan; CARBO = carboplatin * Therapy interrupted after disease progression.

**Table 2:** Inter-observer and inter-method agreement for categorical response assessment.

Methods	κ	95% CI	N of Cases of Disagreement
*Inter-observer agreement*			
1D-RECIST	0.83	0.72–0.94	9
2D-WHO	0.71	0.55–0.86	11
3D-EpSSG	0.86	0.75–0.97	5
3D-Osirix	0.95	0.89–1.00	2
Inter-method agreement			
1D-RECIST/2D-WHO	0.68	0.53–0.83	16
1D-RECIST/3D-EpSSG	0.57	0.43–0.71	24
1D-RECIST/3D-Osirix	0.59	0.45–0.73	23
2D-WHO/3D-EpSSG	0.60	0.45–0.75	20
2D-WHO/3D-Osirix	0.62	0.47–0.77	19
3D-EpSSG/3D-Osirix	0.98	0.93–1.00	1

The 66 patients were divided in four categories (CR = complete response; PR = partial response, SD = stable disease, PD = progressive disease).

**Table 3:** Univariate Cox’s regression models for the two response categories (responders versus non-responders).

Method	Hazard Ratio (95% CI)	*p* Value	Uno’s Concordance Statistic and Standard Error
*Event-free survival*			
1D-RECIST	3.00 (1.15–7.81)	0.0245	0.64 (0.094)
2D-WHO	3.29 (1.31–8.25)	0.0113	0.66 (0.087)
3D-EpSSG	7.09 (2.91–17.25)	<0.0001	0.69 (0.058)
3D-Osirix	5.74 (2.37–13.91)	0.0001	0.68 (0.061)
Overall survival			
1D-RECIST	3.30 (1.05–10.36)	0.0412	0.66 (0.106)
2D-WHO	2.45 (0.87–6.89)	0.0897	0.64 (0.127)
3D-EpSSG	4.34 (1.54–12.26)	0.0055	0.67 (0.074)
3D-Osirix	3.67 (1.30–18.38)	0.0142	0.66 (0.072)

The HR is an estimate of the increase (if >1) or decrease (if <1) in the risk associated with a covariate category versus the reference category (assumed to carry a risk of 1). The larger the HR, the stronger the association with survival.

**Table 4:** Univariate Cox’s regression models for percentage reduction in tumor size.

Method	Hazard Ratio * (95% CI)	*p* Value	Uno’s Concordance Statistic and Standard Error
*Event-free survival*			
1D-RECIST	1.22 (1.05–1.42)	0.0101	0.69 (0.126)
2D-WHO	1.17 (1.06–1.30)	0.0031	0.70 (0.080)
3D-EpSSG	1.17 (1.07–1.29)	0.0007	0.70 (0.092)
3D-Osirix	1.101 (1.03–1.18)	0.0048	0.72 (0.065)
Overall survival			
1D-RECIST	1.12 (0.97–1.28)	0.1213	0.67 (0.15)
2D-WHO	1.11 (0.99–1.24)	0.0684	0.66 (0.11)
3D-EpSSG	1.05 (0.962–1.15)	0.2623	0.68 (0.13)
3D-Osirix	1.07 (0.98–1.15)	0.1475	0.68 (0.11)

* for each 10% reduction in size.

**Table 5:** Multivariate Cox’s regression models for the two response categories adjusted for potential confounders.

Method	Hazard Ratio (95% CI)	*p* Value
*Event-free survival*		
1D-RECIST	3.57 (1.27–10.00)	0.0158
2D-WHO	5.05 (1.66–15.34)	0.0042
3D-EpSSG	14.40 (4.51–46.02)	<0.0001
3D-Osirix	11.60 (3.61–37.29)	<0.0001
*Overall survival*		
1D-RECIST	3.74 (1.07–12.97)	0.0375
2D-WHO	2.77 (0.87–8.87)	0.0849
3D-EpSSG	7.90 (2.05–30.35)	0.0026
3D-Osirix	6.73 (1.74–26.00)	0.0033

**Table 6:** Therapeutic response thresholds according to the 1D-RECIST, 2D- WHO, 3D-EpSSG and 3D-Osirix criteria, based on percentage changes in tumor size after induction therapy.

Response CATEGORY	Measurement Method			
		*1D-Recist*	*2D-WHO*	*3D-EpSSG and 3D-Osirix*
***RESPONDERS***	CR	100% decrease	100% decrease	100% decrease
	VGPR	NA	NA	≥90% but <100% decrease
	PR	≥30% but <100% decrease	≥50% but <100% decrease	≥66% but <90% decrease
	mPR	NA	NA	≥33% but <66% decrease
***NON-RESPONDERS***	SD	Neither PR nor PD	Neither PR nor PD	Neither PR nor PD
	PD	≥20% increase	≥25% increase	≥40% increase

CR = complete response, VGPR = very good partial response PR = partial response, mPR = minor partial response. SD = stable diseasse PD = progressive disease.
